# The Accuracy of Survival Time Prediction for Patients with Glioma Is Improved by Measuring Mitotic Spindle Checkpoint Gene Expression

**DOI:** 10.1371/journal.pone.0025631

**Published:** 2011-10-12

**Authors:** Li Bie, Gang Zhao, Pui Cheng, Gaelle Rondeau, Steffen Porwollik, Yan Ju, Xiao-Qin Xia, Michael McClelland

**Affiliations:** 1 Department of Neurosurgery of the First Clinical Hospital, Jilin University, Changchun, People's Republic of China; 2 Department of Pathology and Laboratory Medicine, University of California Irvine, Irvine, California, United States of America; 3 Vaccine Research Institute of San Diego, San Diego, United States of America; 4 Institute of Hydrobiology, Chinese Academy of Sciences, Wuhan, People's Republic of China; Johns Hopkins Hospital, United States of America

## Abstract

Identification of gene expression changes that improve prediction of survival time across all glioma grades would be clinically useful. Four Affymetrix GeneChip datasets from the literature, containing data from 771 glioma samples representing all WHO grades and eight normal brain samples, were used in an ANOVA model to screen for transcript changes that correlated with grade. Observations were confirmed and extended using qPCR assays on RNA derived from 38 additional glioma samples and eight normal samples for which survival data were available. RNA levels of eight major mitotic spindle assembly checkpoint (SAC) genes (BUB1, BUB1B, BUB3, CENPE, MAD1L1, MAD2L1, CDC20, TTK) significantly correlated with glioma grade and six also significantly correlated with survival time. In particular, the level of BUB1B expression was highly correlated with survival time (*p*<0.0001), and significantly outperformed all other measured parameters, including two standards; WHO grade and MIB-1 (Ki-67) labeling index. Measurement of the expression levels of a small set of SAC genes may complement histological grade and other clinical parameters for predicting survival time.

## Introduction

Glioma tumors are the most common of central nervous system (CNS) neoplasms. The median survival times for low grade (stage I–II) patients average 6–8 years [1], [2] but are only about 3 and 1 year for patients with anaplastic astrocytoma (WHO grade III) and glioblastoma (WHO grade IV), respectively [3], [4], [5].

In a widely used measure, the level of Ki-67 protein is determined using an antibody called MIB-1 in a labeling index (MIB Li) that correlates with aggression and survival time in gliomas [6], [7], [8], [9], [10] and many other cancers [11], [12], [13], [14], [15], [16]. Furthermore, one report indicated that RNA expression of Ki-67 correlated with survival time better than the antibody test, MIB Li, in colorectal carcinoma [17].

In this study, we looked at glioma grades II, III, and IV to uncover additional genes whose RNA expression correlated with grade and survival time. First, we used the statistical analysis suite WebArrayDB (http://www.WebArrayDB.org) [18] to analyze available microarray datasets of glioma RNA expression from various platforms that had already been used to identify hundreds of significant correlations of RNA levels with increasing glioma grade [19], [20], [21], including data stored at the Cancer Genome Anatomy Project (http://cgap.nci.nih.gov/Genes).

We found eight genes involved in the mitotic spindle assembly checkpoint (SAC) which were positively and reliably correlated with grade. SAC proteins are used in chromosome segregation during mitosis [22], [23], [24]. These genes were of interest because an increase in their expression likely implied increased cell division, a phenotype with the potential to correlate with survival in almost any glioma. Crucially, a few of these genes outperformed the current standard for proliferation measurement, Ki-67 (a gene expressed at the same time in the cell cycle [25], [26], [27], [28]), measured either using MIB-1 antibody as a labeling index (MIB Li) or measured at the RNA expression level. Furthermore, these genes had not previously been studied for their behavior in glioma. We used 38 additional glioma samples to further evaluate the association of transcript levels of eight SAC genes with glioma grade and patient survival time.

## Materials and Methods

### Patient samples

Forty four samples, including 38 human glioma specimens (12 for grade II, 13 for grade III, 13 for grade IV) and 6 control samples, were collected from 2003 to 2005 by the Department of Neurosurgery, 1st Affiliated Hospital of Jilin University, China. Tumor tissues were from newly-diagnosed glioma patients, who had received no therapy before sample collection. Samples were collected immediately after surgical resection, snap frozen, and stored at −80°C. Survival time was defined as the period from the date of surgery to the date of death. All surviving patients were followed up for at least five years. All histological diagnoses were made on formalin fixed, paraffin-embedded H&E sections and Ki-67 immunostain, and were categorized according to the 2007 WHO classification, again [29]. Six control samples (normal brain) were obtained from patients undergoing surgery for brain trauma (n = 4) and epilepsy (n = 2). They were reviewed to verify the absence of tumor.

### Ethics statement

Informed written consent was obtained from all patients after an explanation of the study. Samples were collected only when necessary as part of treatment. Only samples in excess of what was needed for pathological assessment were used for research. All samples were coded and handled anonymously. Use of patient material was approved by the Institutional Review Board of Jilin University.

### Cell proliferation evaluated using MIB Li and Mitotic Index

Slides cut from each glioma used in the study were evaluated by two experienced pathologists independently using direct light microscopy. The measurement of Ki-67using the MIB Labeling index (MIB Li) [30] was calculated by determining the percentage of neoplastic cells that were immunopositive. More than 1000 cells were counted in ten separate fields at 400× magnification for each specimen. Only strong nuclear stain was regarded as positive, and weak nuclear or cytoplasmic stain was regarded as negative. The mitotic index (MI) was counted in HE stained slides using scores from 0 to 3, 0: no mitotic figures; 1: ≤2 mitotic figures/field; 2: (3–4) mitotic figures/field; 3: ≥5 mitotic figures/field [6].

### RNA isolation and quality evaluation

Total RNA was isolated using TRIzol Reagent (Invitrogen, CA), following the manufacturer's instructions. The concentration and purity of isolated RNAs were assessed by absorbance (A) readings on a UV spectrophotometer (Hitachi) at 260 and 280 nm, and electrophoresis on a Bio-Rad Experion automated electrophoresis system(Bio-Rad Laboratories, Hercules, CA). The mean ratio value of A260/280 for all RNA samples was 1.80(±0.10); the 28S/18S ratio of samples≥1.5, and no evidence of ribosomal peak degradation was observed.

### Real-time quantitative reverse transcription PCR

Exactly 500 ng total RNA from each sample was used to generate cDNA using SuperScript II Reverse Transcriptase (Invitrogen, Carlsbad, CA). Pairs of PCR primers of 18 to 25 bp in length were designed using AlleleID Version 7.0 software (Premierbiosoft, Palo Alto, CA, **Information S1**). Real-time quantitative PCR (qPCR) was then carried out using an ABI Prism 7900 Sequence Detection System (Applied Biosystems, Carlsbad, CA). A quantity of 15 ng of cDNA was used in a 25 µl PCR reaction containing the appropriate primers and 1×SYBR Green PCR Super mix (BioPioneer, San Diego, CA). Parallel experiments were done using an 18S and HPRT1 primer set [31]. Each sample was run in triplicate, and each PCR experiment included one no-template negative control. The Pfaffl [32] method was used to determine the relative ratio of gene expression for each gene, corrected using expression of two controls by incorporating a normalization factor = (quant[18S]*quant[HPRT1])^1/2^, and referenced to non-neoplasic brain tissues. All primer pairs used in this study had>90% amplification efficiency (**Information S1**).

### Statistical analysis of qPCR data

The qPCR C_T_ values were used to calculate the fold changes of RNA abundance for SAC genes normalized to the reference genes (18S+HPRT1). Before fitting to regression models, the adjusted fold change values for each gene were standardized by subtracting the mean of all values obtained from all samples for each gene and then divided by their standard deviation.

### Differential analysis

For each gene, the differences in expression levels across different WHO grades were tested using one-way ANOVA, and further pair-wise comparisons were made through a simultaneous interface using general parametric models [33].

### Relationship of SAC genes and WHO grades

Linear models were used to explore the relationship between SAC genes and WHO grade. The WHO grades were mapped to numbers using 1 for grade II, 2 for grade III, and 3 for grade IV. These numbers for grades were log-transformed before fitting to the models. Models were fitted to data using leave-one-out cross-validation, i.e., to predict the grade for each of the 38 samples, models were trained with data from the other 37samples. All possible combinations of the eight SAC genes were tested to obtain the models with the most accurate predictions of WHO grade.

### Cox regression analysis

Cox proportional hazards regression was used to model the relationship between survival and all available parameters, including age, sex, WHO grade, tumor diameter, and the level of mitotic checkpoint gene mRNA expression. The optimized model was established from all possible combinations of all variables and factors according to the Akaike information criterion (AIC) [34]. Cox regression was performed using the survival package for the R software environment. Overall survival curves (from diagnosis to death) were obtained using the Kaplan-Meier method. In each survival curve, maximally selected log-rank statistics [35] was used to determine the cutoff-point, an expression value which separates the low-expression samples from the high-expression samples. A *p* value of less than 0.05 was considered statistically significant.

### Prediction of survival time based on SAC genes

34 samples of known survival time (in months) were used to build the linear model for prediction of survival time by SAC genes. Four samples were excluded due to having a last doctor visit but not an exact date of death. The model that best fit the data was selected from all possible combinations using least squares estimates (LSE).

## Results

### Genes chosen for qPCR assays

In order to select genes of potential interest, four independent microarray datasets [19], [20], [36], [37] were used (**Table 1**). These datasets consisted, in total, of 8normal control, 29 grade II, 116 grade III and 618 grade IV gliomas.

**Table 1:** Independent glioma RNA expression microarray datasets.

# of cases	Sample Type	GEO ID	Affymetrix Platform
85	Tumor tissue (frozen)	GSE4412	HG-U133A
265 & 7 controls	Tumor tissue (frozen)	GSE16011	HG-U133Plus2.0
15 & 1 control	Tumor tissue (frozen)	GSE19728	HG-U133Plus2.0
398	Tumor tissue (frozen)	TCGA_GBM	HT_HG-U133A

Data were analyzed on the WebArrayDB cross-platform analysis suite [18] using an ANCOVA model. Genes were sorted in ascending order according to the *p* values for WHO grade, after taking into account gender and patient age as variables. The2000 genes that correlated most significantly with grade were clustered using DAVID [38], [39] and KEGG [39]. Among these most highly ranked genes were at least 25genes associated with cell cycle; for example, TGFβ, MDM2, Smc3, p300, PTTG, HDAC, Cdc6, Cdc14, CDC25A, GADD45, Kip1,2, ORCs, ATMATR, CDK2, CycA, CycB, CycD, MCMs, Wee1, and including four genes that are specifically related to mitotic spindle assembly; BUB1, BUB1B, CDC20, and TTK. Other highly ranked genes included RRM2, FOXM1, p53, and TOP2A. This observation was expected because cancer is a proliferative disease. Expression of these genes is of particular practical interest as markers of progression because it may be altered in most tumors, regardless of the causative mutations. Such markers might be quite reliable for staging tumors despite otherwise massive tumor heterogeneity.

The SAC pathway had not been investigated previously for biomarkers of progression in glioma. Thus, we focused our attention on the SAC genes in this study. We added four additional genes, CENPE, BUB3, MAD1L1 and MAD2L1, which are part of the SAC pathway and highly correlated with glioma grade but were not among the 2000 top genes correlating with grade identified in the microarray survey (**Table 2**).

**Table 2:** Correlation of SAC gene expression with glioma grade using four independent Affymetrix GeneChip datasets.

Gene symbol	synonyms	Sequence Accession ID	Affymetrix Probe Set ID	Gene rank. 22277 probe sets	*p* value*	Description
BUB1	BUB1A/BUB1L/hBUB1	NM_004336	209642_at	1146	1.07E-26	BUB1 budding uninhibited by benzimidazoles 1 homolog
BUB1B	BUBR1/hBUBR1/SSK1	NM_001211	203755_at	1629	2.05E-27	BUB1 budding uninhibited by benzimidazoles 1 homolog beta
BUB3	BUB3L/hBUB3	NM_004725	201456_s_at	6467	2.75E-09	BUB3 budding uninhibited by benzimidazoles 3 homolog
CDC20	CDC20A/p55CDC	NM_001255	205046_at	99	5.24E-27	Cell division cycle 20 homolog
CENPE	KIF10	NM_001813	202870_s_at	3122	7.12E-44	Centromer protein E, 312 kDa
MAD1L1	MAD1/PIG9	NM_003550	204857_at	2469	1.68E-07	MAD1 mitotic arrest deficient-like 1
MAD2L1	MAD2/HSMAD2	NM_002358	203362_s_at	8813	4.22E-19	MAD2 mitotic arrest deficient-like 1
TTK	MSP1/MPS1L1	NM_003318	204822_at	820	1.29E-26	TTK protein kinase
Ki-67	Mki67	NM_002417	212022_at	3820	1.28E-17	antigen identified by monoclonal antibody Ki-67

*p value calculated using ANCOVA.

### RNA expression of SAC genes increases with grade

The expression levels of eight SAC genes were examined in 38 human glioma samples and 6 normal brain tissues by qPCR (**Information S2 and S3**). The 44 samples were classified into four groups: normal, grade II, grade III, and grade IV glioma. For each of the selected genes, the differences in expression levels across the four groups were evaluated using ANOVA. The significance was adjusted for the false discovery (**Table 3**). Not all pairs in this table are significant because the distance between sample classes is different. Normal and grade IV have a large biological difference, and we can see very significant changes for many genes, whereas the difference between normal and grade II is subtle and we find only two genes that rise to the highest level of significance.

**Table 3:** Validation of the mitotic checkpoint genes in glioma tumors by qPCR.

Gene	*P* (ANOVA)	III/II		IV/II		IV/III		II/normal		III/normal		IV/normal	
		Fold	*p*	Fold	*p*	Fold	*p*	Fold	*p*	Fold	*p*	Fold	*p*
BUB1	**1.35E-06**	0.39	1.00E+00	1.01	3.76E-01	0.62	1.00E+00	3.13	**5.46E-04**	3.53	**1.32E-05**	4.14	**8.31E-07**
BUB1B	**4.02E-10**	1.30	**1.18E-03**	2.11	**9.18E-08**	0.81	1.27E-01	0.75	2.43E-01	2.05	**1.22E-05**	2.86	**1.11E-09**
BUB3	**2.08E-02**	0.46	1.00E+00	0.38	9.94E-01	0.08	1.00E+00	1.18	2.39E-01	1.64	**2.89E-02**	1.56	**2.62E-02**
CDC20	**1.66E-13**	1.41	**4.58E-07**	2.14	**3.59E-13**	0.73	**4.97E-02**	0.43	3.95E-01	1.84	**9.73E-08**	2.58	**2.17E-12**
CENPE	**5.78E-04**	0.22	1.00E+00	1.28	6.46E-02	1.06	2.57E-01	1.14	2.43E-01	1.36	6.16E-02	2.42	**3.93E-04**
MAD1L1	**8.76E-03**	0.12	1.00E+00	1.38	1.87E-01	1.26	3.29E-01	0.99	5.01E-01	1.10	3.39E-01	2.37	**8.85E-03**
MAD2L1	**2.08E-02**	0.44	1.00E+00	1.27	2.22E-01	0.83	1.00E+00	0.87	5.81E-01	1.31	2.16E-01	2.14	**1.97E-02**
TTK	**4.80E-05**	0.03	1.00E+00	0.38	9.94E-01	0.41	1.00E+00	2.13	**1.07E-03**	2.10	**4.04E-04**	2.51	**6.66E-05**
Ki-67	**1.35E-06**	1.07	**1.05E-01**	1.71	**6.47E-04**	0.64	8.49E-01	1.06	2.27E-01	2.12	**3.92E-04**	2.76	**1.68E-06**

*p*<0.05 are shown in bold. *p* values were adjusted with FDR.

Overall, expression of the eight SAC genes showed a significant increase with increasing WHO grades (**Table 3**). In contrast to normal samples, overexpression is observed for all genes in grade IV and for most genes, except CENPE, MAD1L1 and MAD2L1, in grade III (*p*<0.05). BUB1 and TTK overexpression in grade II reached statistical significance (*p*<0.05). Further research may indicate if these two genes have potential utility in classifying early stage tumors.

In pairwise comparisons of glioma samples, two genes (BUB1B, CDC20) showed significant difference between grade II and grade IV (*p*<0.05), while expression in grade III does not significantly differ from grade II or grade IV for most genes. This might be explained by the small sample size. The only two exceptions are CDC20 and BUB1B, which changed significantly between all grades (*p*<0.05). To ensure the sample of patients was of adequate size, random groups of only half of the patient samples were tested and all statistically significant observations about the top three genes were supported (data not shown).

We also measuredKi-67 RNA levels because the corresponding protein is currently used to determine a labeling index for glioma [6], [7], [8], [9], [10], [11], [12], [13], [14], [15], [16] and previous work indicated the RNA might be a better prognosticator [17]. Interestingly, Ki-67 RNA level was outperformed by BUB1B and CDC20, overall, and by BUB1 and TTK when applied to grade II gliomas (**Table 3**).

### Identification of WHO grades by SAC genes

Expression of six SAC genes, BUB1, BUB1B, CDC20, CENPE, MAD1L1 and TTK were found to be significantly positively correlated with WHO grades of glioma patients (*p*<0.01, **Table 3**). RNA expression levels of both CDC20 and BUB1B had a Pearson's correlation coefficient with grade of higher than 0.8. After screening all possible combinations of eight SAC genes, we formulated a four-gene model with the highest accuracy in identification of the WHO grades of gliomas (**Information S4**):
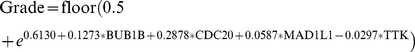
(1)


In which a value of 1 predicts grade II, 2 predicts grade III, and 3 or a greater value predicts grade IV. The values for BUB1B, CDC20, MAD1 L1 and TTK are normalized expression ratios (see Materials and Methods). BUB1B and CDC20 are the most important factors in this model and they have a much bigger weight than MAD2L1. Among the 38 patients used for regression, 34 (89.4%) are classified as expected by this model, and four patients were classified to grades adjacent to observed grades. Using leave-one-out cross-validation, 34 samples were classified in the same grade as estimated by histological examinations by the pathologists, giving 89.4% accuracy, and the other 4 predictions were all adjacent to observed grades (**Information S5**).

CDC20 was the only gene included in all of these 35combinations while the second most frequently occurring gene, BUB1B, was found in 29 of these combinations. This highlights the possibly useful role of CDC20 and BUB1B in estimating grades of glioma samples.

### Multivariate analysis of the correlation between prognostic parameters and survival time

Cox multivariate proportional hazards models with different combinations of factors were obtained for the 38 patient samples where survival time was known (**Table 4**).

**Table 4:** Multivariate analysis on survival time.

Model	Parameter	Risk ratio	95% CI	*p*
1. Cox regression without genes	Sex (male vs female)	0.35	0.60–2.63	0.56
	Age	0.53	0.98–1.04	0.47
	WHO (Grade III vs Grade II)	1.08	0.19–1.03	0.59
	WHO (Grade IV vs Grade II)	3.56	0.07–0.49	0.01*
2. Cox regression with genes	Sex (male vs female)	0.57	0.19–1.72	0.32
	Age	1.03	0.99–1.07	0.12
	WHO (Grade III vs Grade II)	0.43	0.06–3.10	0.58
	WHO (Grade IV vs Grade II)	0.19	0.01–2.79	0.23
	BUB1	1.07	0.54–2.12	0.84
	BUB1B	7.38	1.72–31.65	0.01*
	BUB3	0.88	0.54–1.44	0.62
	CDC20	0.60	0.15–2.32	0.46
	CENPE	1.30	0.69–2.46	0.41
	MAD1L1	0.73	0.32–1.64	0.44
	MAD2L1	1.58	0.66–3.77	0.30
	TTK	0.65	0.31–1.34	0.24
	MIB Li (Ki-67 antibody)	1.08	0.98–1.21	0.13
	MI	1.32	0.45–3.85	0.61
3. Optimized Cox regression	BUB1B	10.98	2.87–17.10	1.90E-05*
	CDC20	0.38	0.20–0.69	1.69E-03*
	MIB Li (Ki-67 antibody)	1.57	1.02–1.14	4.72E-03*

Asterisk (*) for *p*<0.01, n = 38.

In the first model in **Table 4**, which excluded SAC gene expression data, WHO grade were significantly correlated with survival time, but age and sex were not. In models that included SAC gene expression (model 2 and model 3) grades no longer added information and only BUB1B was identified as a significant factor with the highest risk ratio (*p*<0.01). Indeed, even Ki-67 protein as measured by the labeling index (MIB Li) and mitotic index (MI), which significantly correlate with survival time (**Information S6**), did not add additional information when SAC genes were included (model 2). In model 3 each combination of factors is examined to determine the minimum number of factors that capture the most information about survival time. Only factors that add any information to the prediction are retained. BUB1B dominated this regression, with small adjustments from CDC20 and from MIB Li. Thus, it appears that BUB1B is an independent prognostic factor for survival time.

### Prediction of survival time using SAC gene expression

By applying linear regression to all possible gene combinations, we obtained a four-gene combination with the minimal least squares estimate (LSE, 34.96) in leave-one-out cross-validation: BUB1, BUB1B, CENPE, and TTK (**Information S7**). The regression equation for these patients is:
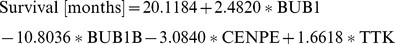
(2)


**Figure 1:**
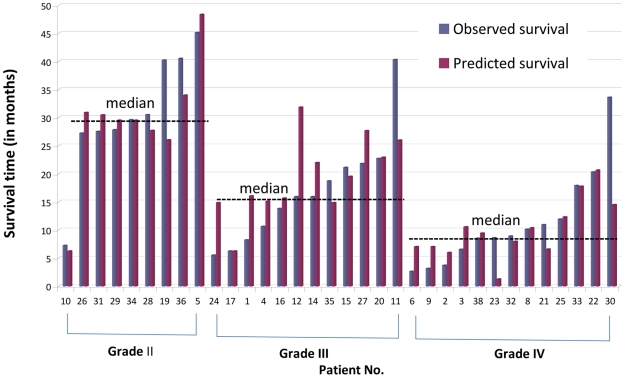
Observed versus predicted survival times using the four gene model.

The predicted months of survival closely matched the observed values for most patients (Four-Gene Model in **Figures 1** **,** **2**). Coefficients used in cross-validation and prediction for each sample is listed in **Information S8**. Among the four genes used in this model, the coefficient for BUB1B was much higher than that of the other genes, which consequently contributed little additional information.

**Figure 2:**
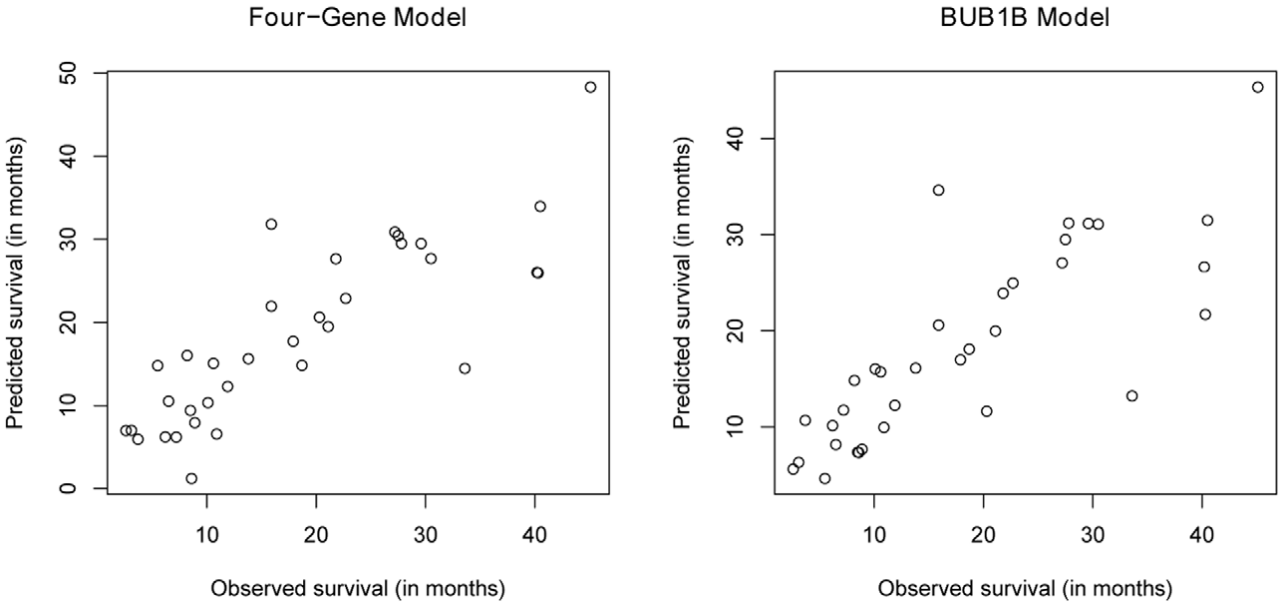
Scatter plots of observed and predicted survival times using leave-one-out cross-validation on 34 deceased glioma patients.

BUB1B is the only gene that appeared in all gene combinations that can be used to make survival time predictions with the lowest least square errors (LSE) (**Information S9**). Thus, we also fitted BUB1B alone to a regression model:
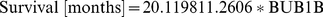
(3)


This single gene model obtains a LSE of 45.64 in cross-validation, and the plots of predicted versus observed survival time look very similar to the plots using the four-gene model in equation (2) (**Figure 2**).

### Kaplan-Meier survival curves for BUB1B and MIB Li

**Figure 3:**
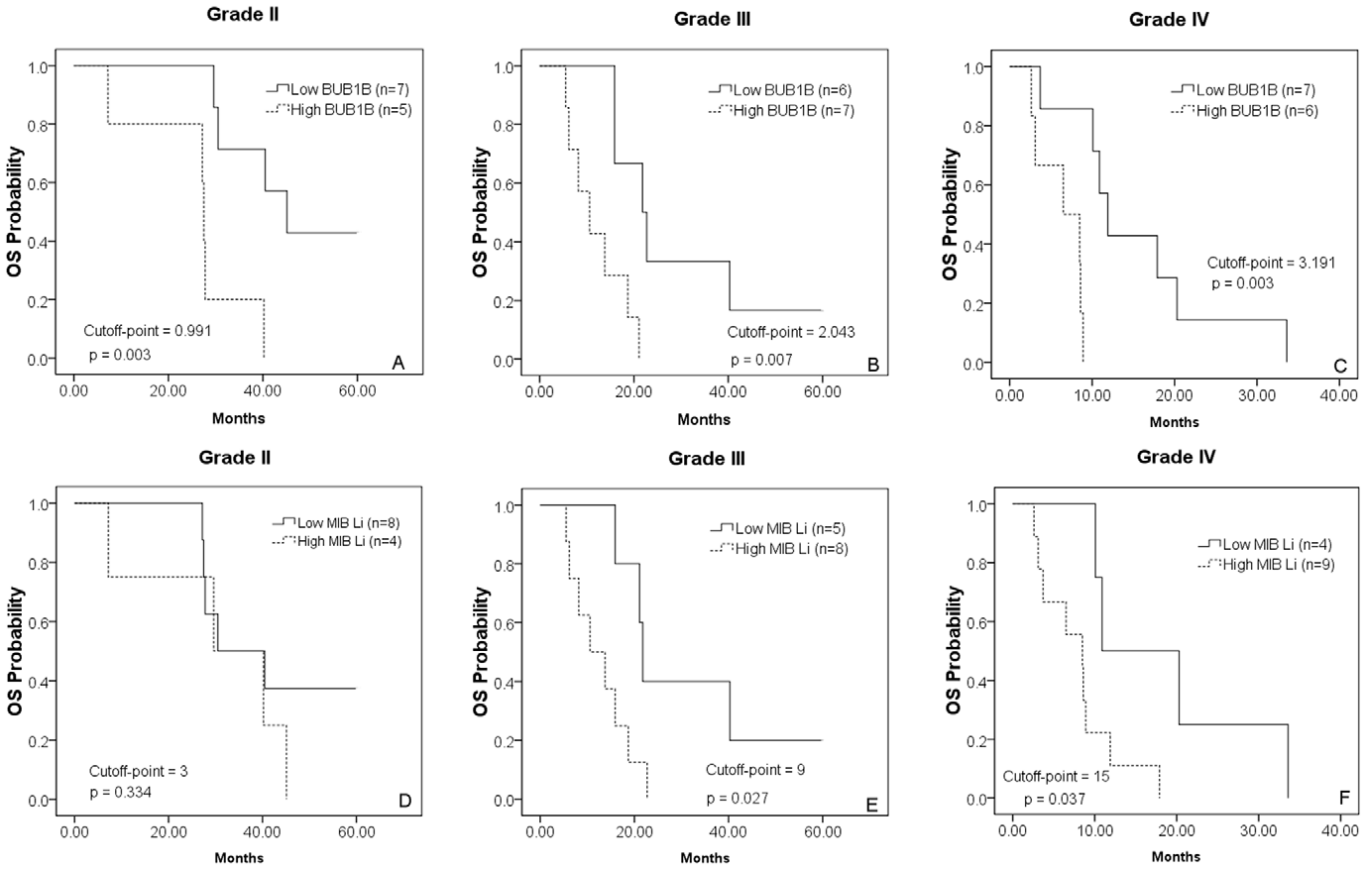
Kaplan-Meier estimates of overall survival time. Panels A, B, and C use BUB1BRNA expression as determined by qPCR. Panels D, E, and F use the MIB labeling index (MIB Li) that measures Ki-67 protein. Log-rank statistics is used to determine the cutoff-point, which is the expression value that divides low-expression samples from high-expression samples. OS = Overall survival.

In the analysis presented in **Figure 3**, six charts are plotted using grade II, grade III, and grade IV with separate curves for low BUB1B levels, high BUB1B levels, low MIB Li and high MIB Li. The cutoffs points within each grade are selected to maximize discrimination in each set of data. If there were no correlation between BUB1B expression or MIB Li and survival time then no significant cutoff would be found. All BUB1B expression charts display significantly different survival time between low expression BUB1B samples and high expression BUB1B samples (*p*<0.01) in three WHO grade. Thus, the expression level of BUB1B reveals information on the survival probability for glioma patients beyond that revealed by grade alone. In contrast, MIB Li showed little or no significant difference for any grade and was outperformed by BUB1B for all grades.

### SAC protein levels increases with grade

The Human Protein Atlas (HPA, http://www.proteinatlas.org/) collects information on proteins detected by antibodies in a wide variety of tissue samples arranged as tissue microarrays, including gliomas. The result of immunohistochemistry for antibodies directed against seven of the SAC proteins in our study (all except CENPE) are available for 11 grade III/IV glioma and three normal brain cores in the HPA database. The level of staining by each antibody was scored by board certified pathologists as: negative, weak, moderate, or strong. Example images are shown in **Information S10**. We translated the four different categories to 0, 1, 2, and 3 to permit analysis using a Wilcox rank sum test. The demarcations of scoring categories are imprecise and subjective. Nevertheless, antibody stains for proteinsBUB1, BUB1B, BUB3, CDC20, and TTK were at significantly higher levels in high grade glioma than in normal brain by this test (**Table 5**). This data is consistent with the higher mRNA expression levels we observed in both grade III and grade IV for these five genes compared to normal brains. Antibody staining of two proteins, MAD1L1 and MAD2L1, appeared to experience less increase with grade, which is also consistent with our observation that for these two genes RNA overexpression was significant relative to normal tissue only in grade IV and not in grade III (**Table 3**).

**Table 5:** Wilcox rank sum test of proteins between normal samples and malignant glioma samples.

Proteins	Normal samples			Grade III+IV glioma samples				*p*
	Negative	Weak	Moderate	Negative	Weak	Moderate	Strong	
BUB1	0	0	3	0	0	4	7	**0.036** *
BUB1B	0	3	0	0	0	10	2	**0.001** *
BUB3	0	0	3	0	0	4	7	**0.036** *
CDC20	3	0	0	4	1	6	0	**0.041** *
MAD1L1	0	0	3	0	2	4	6	0.193
MAD2L1	3	0	0	9	1	0	0	0.358
TTK	0	3	0	1	2	8	0	**0.038** *

*p<0.05. Examples of immunohistochemical stains are shown in Information S10.

## Discussion

WHO grade of gliomas is currently the major indicator used for prognosis and for treatment protocols. The 2007 WHO grading system [29] uses histopathological characteristics such as brisk mitotic activity (Ki-67: MIB Li) [40], increased cellularity, necrosis, and frequent invasion of brain parenchyma to classify gliomas into four broad grades. Sophisticated methods of assessing survival based on other clinical parameters have also been developed [41].

In combination with the current clinical methods, gene expression profiles are potentially powerful predictors of survival [19], [20], [21] with additional potential as markers for diagnosis, as a guide to therapy, and even as potential therapeutic targets. Genetic analyses over the past several years have defined the major targets that are associated with the formation of glioma including EGRF, PTEN, TP53, IDH1/2, CDKN2A, MGMT, and others [42], which likely have downstream effects on other genes to improve tumor survival. For example, p53 inhibits tumor cell growth through the indirect regulation of CDC20, one of the genes of interest in this report [43]. While the lesions involved in causing progression in glioma may vary from patient to patient, these changes likely converge on a few critical regulatory pathways. Any expression changes in these common pathways could prove useful in prognosis because the changes may occur in most tumors, regardless of the prime movers in disease progression in any particular tumor.

We further investigated hundreds of microarray profiles of glioma and a few normal brain tissues that had previously been used successfully to predict survival time in gliomas [19], [20], [21]. Our goal was to identify a small subset of markers that could reliably substitute for or improve uponKi-67 measurements using MIB Li [6], [7] or multigene expression profiles, and then to confirm these markers on independent samples.

To identify pathways that might undergo changes in RNA expression with increasing grade using the WebarrayDB cross-platform analysis suite [18]. Among a number of different groups of functionally related genes that correlated with grade, one group contained genes associated with the spindle assembly checkpoint (SAC). Importantly, if these genes were to be of potential clinical utility, expression of at least two of these SAC genes appeared to correlate better with grade than did Ki-67 RNA (**Table 2**). The SAC is involved in accurate chromosome segregation between two daughter cells during mitosis. There are already data in the literature that suggest that SAC gene expression may be associated with aggressiveness of cancers. Studies of malignant bladder cancer and breast cancer suggested that increased mRNA levels of mitotic checkpoint genes are correlated with tumor progression [44], [45]. A strong correlation between expression of BUB and gastric cancer cell proliferation has been observed [46]. Combined expression of BUB1B and PINK1 was the best predictor of overall survival in adrenocortical tumors by microarray [47]. Many immunohistochemistry investigations have shown that overexpression of BUBR1, the protein of BUB1B, significantly correlates with higher histological grade, advanced pathological stage, and high cell proliferation in different types of tumors, e.g. with tumor recurrence and disease progression in bladder cancer [44]; with deep invasion, lymph node metastasis, liver metastasis, and poor prognosis in gastric cancer [48]; and with advanced stage, serous histology and high grade in ovarian cancer [49]. On the other hand, a lower level of BUBR1 correlated with low recurrence-free survival rates in ovarian cancer [49] and aneuploidy in colorectal cancer [50]. BUBR1 was used as an independent predictor for poor prognosis in pancreatobiliary-type tumors by tissue microarray [51]. However, correlations of SAC gene expression with aggressiveness have not been reported for glioma except for CDC20 that has higher expression in glioblastoma than in low grade glioma [52].

To validate the RNA expression changes in SAC genes in the microarray data (**Table 2**), we performed qPCR analysis on RNA from six additional normal brain samples and 38 additional gliomas that had survival time data. The eight SAC genes were significantly overexpressed at the RNA level in glioblastomas (grade IV) in comparison to controls (**Table 3**), and all were almost monotonically increased in expression along with grade, indicating that they might serve as prognostic glioma markers. Two genes, BUB1B and CDC20, outperformed Ki-67 RNA. This is consistent with past reports for colorectal carcimona in whichMIB-1 Li had no significant correlation with Ki-67 mRNA expression [17], [53]

BUB1 and TTK were significantly differentially expressed between low grade gliomas and normal brain tissues (*p*<0.01, **Table 3**). Further research will indicate if these two genes have utility in classifying early stage tumors. These genes have previously been associated with cancer though not glioma; BUB1 mutation was associated with lymph node metastasis and shorter relapse-free survival after surgery in colorectal cancers [54], TTK had an increased expression level in anaplastic thyroid carcinoma [55], and TTK expression correlated with tumor node metastasis (TNM) stage in gastric cancer [56].

We used gene expression of SAC genes to build models that correlate with grade. A4-gene regression model (Equation 1) was sufficient to generate 92.1% identity with grade. Considering the potential bias in identification of grades by histological diagnoses and the arbitrary boundaries between adjacent grades, this performance is extremely good.

In a multivariate proportional hazards model without genes (Model 1 in **Table 4**) WHO grade is identified as a significant factor for survival (*p*<0.05). However, WHO grade adds no power to classification when SAC gene expression profiles are added to the model (Model 2 in **Table 4**). MIB labeling index and mitotic index (MI) also add no additional information in model 2. WHO grade is not present when this model is optimized to identify factors that best predict outcome (Model 3 in **Table 4**). Although MIB Li is retained in this model, it is a minor factor compared to BUB1B expression. Thus, qPCR assays of SAC genes, particularly BUB1B, might be used as an objective complement to histological diagnosis for identification of glioma grades, MIB Li, MI, and the multigene RNA profiles that have been proposed [19], [20], [21].

**Figure 3** illustrates that BUB1B was able to distinguish among grade IV samples with significance (*p* = 0.003). Two previous studies are used microarray data to subclassify glioblastomas (grade IV) [57], [58]. The SAC genes were not prominent among these genes. However, given that our study encompassed all grades, this lack of concordance is not surprising. We will need larger datasets in order to validate whether we are able to define subclasses of glioblastoma using just SAC genes. In the future, it will be worth studying whether all three sets of predictors can be combined into an even more powerful predictor, at least for grade IV glioblastomas.

Our model of BUB1B RNA expression provided median survival estimates very close to the observed median survival rate for the WHO grades in leave-one-out cross validation. There are inevitably some outliers in any model of clinical data (**Figure 2**) because the models cannot take into account other important factors such as differences in sample sources, treatments, genes in other pathways, and other potential biological factors. This issue can be addressed by using samples with more detailed follow-up examinations and qPCR assays for more related genes. It is notable how few outliers are seen even without this additional information.

Why do SAC gene expression levels increase with increasing grade in gliomas? The simplest explanation is that expression is simply correlated with the rate of cell division, which is, in turn correlated with survival time.

Another possibility is that increased SAC gene expression is a homeostatic response to defects in other molecular components. The mitotic spindle assembly checkpoint ensures that cells with defective mitotic spindles or defective interaction between the spindles and kinetochores do not initiate chromosomal segregation during mitosis. The SAC can protect the cell from chromosome mis-segregation and aneuploidy during cell division [59], [60]. Increased chromosomal instability is a major driving force for tumor development and progression [28], [54], [61]. In general, tumor cells become increasingly aneuploid with tumor progression [62], [63]. Increased SAC gene expression is correlated with aneuploidy in breast cancer [45]. Previous studies have proven that defects in the mitotic checkpoint might contribute to tumorigenesis [59], [64]. However, total loss of checkpoint gene function can be catastrophic even for cancer cells [65], making the SAC a potentially interesting target for therapy in brains, where the side effect of inhibiting cell division may have little consequence.

Another possible mechanism for the changes in expression we observed would be mutations in one or more SAC gene. To date, only one study has found such mutations; BUB1 mutation is associated with lymph node metastasis and shorter relapse-free survival after surgery in colorectal cancers [54]. In contrast, studies have failed to find mutations in BUB1, BUB1B and BUB3 as a significant causation of chromosomal instability in glioblastomas [66]. Mutations were not found in mitotic checkpoint genes in breast cancer [45], bladder cancer [67] or gastric cancer [68]. Thus, change in SAC gene expression could be due to lesion in other genes that act to increase cell division. A search for epigenetic changes in SAC genes may be fruitful.

Preliminary immunohistochemistry evidence indicates that the proteins encoded by the SAC genes investigated here are also induced in gliomas, with BUB1B again being the most significant (**Table 5**). Thus immunohistochemistry might be useful as an alternative to qPCR as a prognostic assay. Furthermore, the spatial resolution of immunohistochemistry within cells or across a tumor might identify tumors where only a portion is highly aggressive, leading to a more accurate survival time prediction compared to qPCR-based estimates from bulk samples.

A single marker such as BUB1B will not capture all the variability in subtypes of glioma. Instead, such a gene may be useful in combination with other markers. Over the past decade, there has been an increasing use of molecular markers in the assessment and management of glioma patients [69]. For example, the methylation of MGMT has been shown to be useful as a prognostic biomarker in some circumstances [70]; EGFR vIII expression enables identification of a subgroup of tumors with more aggressive behavior [71]; and IDH1/IDH2 mutations have strong prognostic value in grade III astrocytomas and in glioblastomas [72]. A recent study identified a classifier based on the RNA levels of nine genes that has potential value for therapy optimization in glioblastoma (grade IV), the most advanced form of glioma [57]. Another report divided glioblastoma into different subtypes using cluster analysis of microarray expression data from the literature [58]. Such differentiation into subclasses could lead to different therapy strategies [42] as well as better clinical trial designs.

In conclusion, two SAC genes, BUB1 and TTK, showed increased RNA expression compared to normal brain even in the lowest grades of glioma, perhaps indicating their future utility for differentiating among low grade gliomas. Another SAC gene, BUB1B is highly correlated with survival time, outperforming other markers, including grade and Ki-67 mRNA level. Measuring the expression of BUB1B gene might be a useful addition to the repertoire of clinicians for staging gliomas. This ability to use just one or a small handful of genes to predict outcome could have an impact on clinical trials where matching patients across treatment arms more accurately would lead to a considerable increase in power.

## Supporting Information

Information S1 **Primer sequences and amplification summary.**
(DOC)

Information S2 **Patient clinical information and raw qPCR data.**
(DOC)

Information S3 **Patient clinical information and normalized qPCR data.**
(DOC)

Information S4 **Screening all possible combinations of eight SAC genes to construct a linear model to predict grade.**
(DOC)

Information S5 **Leave-one-out cross-validation for prediction of WHO grade for 38 patients.**
(DOC)

Information S6 **Correlation analysis between SAC genes and other factors in 38 glioma samples.**
(DOCX)

Information S7 **Screening all possible combinations of eight SAC genes to construct a linear model to predict survival time.**
(DOC)

Information S8 **Leave-one-out cross-validation of prediction of survival time for 34 deceased patients using the four-gene model.**
(DOC)

Information S9 **Leave-one-out cross-validation of prediction of survival time for 34 deceased patients using the one gene BUB1B model.**
(DOC)

Information S10 **Examples of immunohistochemical staining.**
(DOC)
