# Supplementary material for: The Accuracy of Survival Time Prediction for Patients with Glioma Is Improved by Measuring Mitotic Spindle Checkpoint Gene Expression
Source: PLoS One. 2011 Oct 12;6(10):e25631. doi: 10.1371/journal.pone.0025631 (PMC3192043; doi:10.1371/journal.pone.0025631)
Supplement: Information S1 — Primer sequences and amplification summary. (DOC) [file pone.0025631.s001.doc]

**Supporting Information S1. Primer sequences and amplification summary**

| | Gene | Primer sequence [5’→ 3’] | Amplification size (bp) | Amplification efficiency (%) | | --- | --- | --- | --- | | 18S rRNA | F: GGAGTATGGTTGCAAAGCTGA | 129 | 96% | |  | R: ATCTGTCAATCCTGTCCGTGT |  |  | | HPRT1 | F: TGAGGATTTGGAAAGGGTG | 118 | 103% | |  | R: GAGCACACAGAGGGCTACAA |  |  | | BUB1 | F: AAAGAGTCAAATATGGAACGAAGA | 98 | 102% | |  | R: ACAACCTGCTCAACATCAAC |  |  | | BUB1B | F: CAGCAGAAACGGGCATTT | 74 | 107% | |  | R:TACCTATCCCAAACATCCAGAG |  |  | | BUB3 | F: AACAAGCAGGGTTATGTATTAAGC | 93 | 98% | |  | R: GGCATACTTCTTCTTCTGTACCT |  |  | | CENPE | F: GCTGATCTCACAGAAGAAGTTG | 86 | 110% | |  | R: TCTCCATAATGCCTGCTCTT |  |  | | MAD1L1 | F: GGAGCTGGAGAACGAGAG | 92 | 96% | |  | R: GGTCTTCTGGAGTCCTGATG |  |  | | MAD2L1 | F: CACTGTTGGAAGTTTCTTGTTCA | 74 | 93% | |  | R: ACTGTGGTCCCGACTCTT |  |  | | CDC20 | F: GAACTCAAAGGTCACACATCC | 96 | 98% | |  | R: TAGCCTCAGGGTCTCATCT |  |  | | TTK | F: ATTGTGATGTGAAGACAGATGATT | 84 | 108% | |  | R: CAACCAAATCTCGGCATTCT |  |  | | Ki-67 | F: ATTGATCGTTCCTTCAGGTATG | 134 | 96% | |  | R: TCATCAGGGTCAGAAGAGAAG |  |  | |
| --- | --- | --- | --- | --- | --- | --- | --- | --- | --- | --- | --- | --- | --- | --- | --- | --- | --- | --- | --- | --- | --- | --- | --- | --- | --- | --- | --- | --- | --- | --- | --- | --- | --- | --- | --- | --- | --- | --- | --- | --- | --- | --- | --- | --- | --- | --- | --- | --- | --- | --- | --- | --- | --- | --- | --- | --- | --- | --- | --- | --- | --- | --- | --- | --- | --- | --- | --- | --- | --- | --- | --- | --- | --- | --- | --- | --- | --- | --- | --- | --- | --- | --- | --- | --- | --- | --- | --- | --- | --- | --- | --- | --- |
