# Supplementary material for: The Accuracy of Survival Time Prediction for Patients with Glioma Is Improved by Measuring Mitotic Spindle Checkpoint Gene Expression
Source: PLoS One. 2011 Oct 12;6(10):e25631. doi: 10.1371/journal.pone.0025631 (PMC3192043; doi:10.1371/journal.pone.0025631)
Supplement: Information S2 — Patient clinical information and raw qPCR data. (DOC) [file pone.0025631.s002.doc]

**Supporting Information S2. Patient clinical information and raw qPCR data**

| **No.** | **18S** | **HPRT1** | **BUB1** | **BUB1B** | **BUB3** | **CENPE** | **MAD1L1** | **MAD2L1** | **CDC20** | **TTK** | **Ki-67** | **Age(years)** | **Survival (months)** | **Alive (0)** | **Gender** | **Histology Classification** | **WHO Grade** | **MIB Li** | **MI** |
| --- | --- | --- | --- | --- | --- | --- | --- | --- | --- | --- | --- | --- | --- | --- | --- | --- | --- | --- | --- |
| 1 | 8.952 | 25.613 | 26.697 | 23.096 | 23.51 | 27.877 | 25.563 | 24.563 | 24.968 | 23.946 | 21.886 | 45 | 8.20 | 1 | M | AA | III | 15 | 2 |
| 2 | 7.304 | 23.932 | 23.703 | 21.134 | 20.733 | 23.247 | 19.151 | 19.651 | 22.666 | 21.884 | 19.534 | 65 | 3.70 | 1 | M | MIXED | IV | 22 | 3 |
| 3 | 9.708 | 27.077 | 25.318 | 23.557 | 24.341 | 26.037 | 23.393 | 23.893 | 25.351 | 22.707 | 22.262 | 53 | 6.50 | 1 | M | AO | IV | 41 | 3 |
| 4 | 8.201 | 25.422 | 23.697 | 22.744 | 21.634 | 24.005 | 20.77 | 21.27 | 24.583 | 21.744 | 21.154 | 55 | 10.6 | 1 | F | AO | III | 14 | 2 |
| 5 | 12.332 | 26.784 | 27.471 | 28.015 | 25.233 | 29.446 | 26.936 | 26.936 | 30.279 | 25.165 | 27.437 | 41 | 45.1 | 1 | F | AO | II | 3 | 0 |
| 6 | 8.285 | 25.96 | 23.397 | 22.115 | 20.938 | 25.252 | 21.948 | 21.448 | 23.974 | 23.115 | 20.765 | 39 | 2.60 | 1 | M | GBM | IV | 18 | 3 |
| 7 | 8.352 | 25.558 | 26.635 | 23.307 | 23.939 | 27.673 | 23.825 | 24.325 | 24.891 | 23.307 | 23.113 | 44 | 60.3 | 0 | M | AA | III | 2 | 1 |
| 8 | 12.833 | 28.341 | 29.554 | 26.318 | 27.507 | 28.354 | 26.315 | 25.315 | 27.772 | 26.718 | 25.868 | 55 | 10.1 | 1 | M | MIXED | IV | 11 | 1 |
| 9 | 8.352 | 25.385 | 23.476 | 21.931 | 22.44 | 25.479 | 22.301 | 20.301 | 24.562 | 23.531 | 20.530 | 35 | 3.10 | 1 | M | GBM | IV | 27 | 3 |
| 10 | 8.054 | 25.791 | 26.827 | 22.49 | 22.653 | 26.522 | 24.331 | 25.331 | 26.828 | 24.49 | 21.639 | 34 | 7.20 | 1 | F | AO | II | 15 | 1 |
| 11 | 8.5 | 26.341 | 24.513 | 23.944 | 23.938 | 27.757 | 23.636 | 23.136 | 24.74 | 23.184 | 22.894 | 47 | 40.3 | 1 | F | AO | III | 6 | 0 |
| 12 | 15.083 | 28.374 | 29.808 | 28.922 | 28.438 | 29.834 | 26.064 | 27.064 | 30.19 | 27.622 | 29.279 | 21 | 15.9 | 1 | F | AA | III | 8 | 2 |
| 13 | 10.602 | 27.748 | 24.978 | 27.057 | 26.003 | 27.78 | 28.85 | 27.85 | 27.527 | 24.607 | 25.405 | 2 | 60.6 | 0 | M | AO | II | 0 | 0 |
| 14 | 12.361 | 28.948 | 27.382 | 26.813 | 25.468 | 28.426 | 25.409 | 26.409 | 28.644 | 25.563 | 26.263 | 51 | 15.9 | 1 | M | AO | III | 16 | 2 |
| 15 | 10.377 | 27.449 | 26.27 | 25.133 | 24.023 | 27.197 | 24.662 | 23.162 | 26.627 | 24.633 | 24.450 | 23 | 21.1 | 1 | M | AA | III | 7 | 1 |
| 16 | 7.563 | 25.24 | 24.466 | 22.406 | 23.072 | 25.892 | 24.027 | 23.027 | 23.903 | 23.156 | 20.842 | 37 | 13.8 | 1 | M | AA | III | 12 | 2 |
| 17 | 10.384 | 26.341 | 30.467 | 29.394 | 29.378 | 29.337 | 23.611 | 24.611 | 30.763 | 28.044 | 30.195 | 55 | 6.20 | 1 | F | AO | III | 19 | 2 |
| 18 | 11.505 | 27.889 | 29.63 | 27.951 | 25.028 | 29.654 | 24.475 | 25.475 | 29.41 | 25.901 | 26.400 | 40 | 60.5 | 0 | M | AO | II | 1 | 0 |
| 19 | 9.53 | 27.099 | 26.634 | 25.245 | 25.683 | 27.506 | 26.132 | 25.132 | 27.074 | 24.265 | 24.196 | 43 | 40.2 | 1 | M | AO | II | 5 | 1 |
| 20 | 11.497 | 27.222 | 25.65 | 25.983 | 26.67 | 26.854 | 26.722 | 26.222 | 27.77 | 25.683 | 25.732 | 42 | 22.7 | 1 | M | AA | III | 9 | 1 |
| 21 | 9.462 | 26.158 | 25.092 | 23.175 | 26.204 | 25.95 | 24.013 | 24.013 | 25.001 | 24.775 | 24.910 | 56 | 10.9 | 1 | M | GBM | IV | 6 | 0 |
| 22 | 10.321 | 27.589 | 25.751 | 24.445 | 26.878 | 29.07 | 25.559 | 28.059 | 25.998 | 23.445 | 23.946 | 53 | 20.3 | 1 | M | GBM | IV | 13 | 1 |
| 23 | 12.095 | 28.723 | 30.112 | 25.395 | 27.557 | 28.304 | 25.934 | 28.934 | 27.34 | 25.645 | 24.331 | 49 | 8.60 | 1 | F | GBM | IV | 26 | 2 |
| 24 | 8.601 | 26.857 | 24.837 | 22.635 | 22.403 | 28.274 | 24.162 | 23.162 | 24.982 | 23.135 | 20.835 | 36 | 5.50 | 1 | M | AO | III | 21 | 2 |
| 25 | 11.895 | 28.546 | 27.731 | 25.656 | 26.337 | 27.47 | 25.636 | 24.636 | 27.293 | 24.326 | 24.575 | 41 | 11.9 | 1 | F | GBM | IV | 32 | 3 |
| 26 | 9.968 | 26.739 | 24.813 | 25.244 | 23.012 | 28.084 | 25.181 | 25.181 | 27.156 | 24.994 | 24.489 | 59 | 27.2 | 1 | M | AO | II | 2 | 0 |
| 27 | 13.283 | 28.992 | 28.699 | 27.567 | 26.65 | 30.278 | 28.764 | 29.264 | 29.686 | 25.967 | 27.018 | 57 | 21.8 | 1 | F | AA | III | 7 | 2 |
| 28 | 13.697 | 29.277 | 28.789 | 28.553 | 26.997 | 28.573 | 27.348 | 26.848 | 30.084 | 26.653 | 28.557 | 30 | 30.5 | 1 | F | AO | II | 1 | 0 |
| 29 | 12.408 | 28.883 | 30.255 | 27.759 | 28.013 | 29.656 | 28.345 | 28.845 | 29.748 | 25.712 | 27.011 | 57 | 27.8 | 1 | M | AO | II | 2 | 1 |
| 30 | 9.677 | 27.048 | 25.222 | 24.064 | 22.616 | 26.742 | 23.553 | 24.053 | 25.7 | 23.813 | 23.519 | 54 | 33.6 | 1 | M | AO | IV | 12 | 2 |
| 31 | 9.732 | 26.758 | 27.73 | 25.354 | 25.725 | 28.71 | 26.278 | 25.778 | 27.676 | 24.204 | 24.243 | 30 | 27.5 | 1 | M | AO | II | 2 | 1 |
| 32 | 11.675 | 28.6 | 25.234 | 25.172 | 24.711 | 26.466 | 24.241 | 24.241 | 27.259 | 25.148 | 24.381 | 31 | 8.90 | 1 | M | GBM | IV | 31 | 3 |
| 33 | 12.21 | 28.293 | 28.072 | 26.114 | 26.28 | 29.296 | 27.052 | 27.052 | 28.087 | 26.113 | 25.672 | 20 | 17.9 | 1 | F | AO | IV | 15 | 2 |
| 34 | 10.206 | 27.292 | 26.855 | 25.984 | 25.756 | 28.408 | 23.983 | 24.483 | 28.896 | 25.994 | 25.039 | 36 | 29.6 | 1 | M | AO | II | 4 | 1 |
| 35 | 9.546 | 26.626 | 26.072 | 24.179 | 24.341 | 27.426 | 26.226 | 25.726 | 26.382 | 25.779 | 23.332 | 10 | 18.7 | 1 | M | AO | III | 15 | 2 |
| 36 | 12.702 | 29.288 | 29.25 | 28.213 | 27.406 | 30.355 | 26.38 | 27.88 | 30.04 | 25.613 | 27.580 | 34 | 40.5 | 1 | M | AO | II | 1 | 0 |
| 37 | 10.434 | 27.071 | 27.159 | 26.806 | 25.552 | 27.604 | 25.462 | 24.962 | 28.74 | 24.256 | 25.408 | 5 | 60.9 | 0 | M | AO | II | 0 | 0 |
| 38 | 9.757 | 26.805 | 25.374 | 23.391 | 23.968 | 27.331 | 24.978 | 24.478 | 25.613 | 24.391 | 22.596 | 45 | 8.50 | 1 | M | GBM | IV | 16 | 2 |
| Normal1 | 11.807 | 28.908 | 30.651 | 29.819 | 27.911 | 30.51 | 27.989 | 28.489 | 29.845 | 30.019 | 27.568 | 23 |  |  | F | Normal |  |  |  |
| Normal2 | 11.478 | 28.927 | 32.164 | 27.795 | 28.113 | 29.641 | 27.508 | 27.008 | 30.191 | 27.345 | 27.800 | 34 |  |  | M | Normal |  |  |  |
| Normal3 | 12.154 | 28 | 31.219 | 29.545 | 28.763 | 30.915 | 28.328 | 28.828 | 30.387 | 29.28 | 28.544 | 59 |  |  | M | Normal |  |  |  |
| Normal4 | 12.42 | 29.503 | 32.308 | 27.747 | 28.183 | 31.353 | 28.599 | 29.099 | 30.394 | 27.497 | 29.950 | 12 |  |  | F | Normal |  |  |  |
| Normal5 | 11.82 | 28.919 | 32.31 | 28.196 | 29.22 | 30.411 | 29.246 | 28.746 | 30.625 | 28.496 | 28.692 | 29 |  |  | F | Normal |  |  |  |
| Normal6 | 11.602 | 28.85 | 31.736 | 27.558 | 27.584 | 30.872 | 28.259 | 27.759 | 29.765 | 27.568 | 27.609 | 47 |  |  | M | Normal |  |  |  |

* 0 = alive, 1 = dead.

** AO: astrocytomas, AA: anaplastic astrocytomas, GBM: glioblastoma
