# Supplementary material for: The Accuracy of Survival Time Prediction for Patients with Glioma Is Improved by Measuring Mitotic Spindle Checkpoint Gene Expression
Source: PLoS One. 2011 Oct 12;6(10):e25631. doi: 10.1371/journal.pone.0025631 (PMC3192043; doi:10.1371/journal.pone.0025631)
Supplement: Information S3 — Patient clinical information and normalized qPCR data. (DOC) [file pone.0025631.s003.doc]

**Supporting Information S3. Patient clinical information and normalized qPCR data.**

| No. | 18S | HPRT1 | BUB1 | BUB1B | BUB3 | CENPE | MAD1L1 | MAD2L1 | CDC20 | TTK |
| --- | --- | --- | --- | --- | --- | --- | --- | --- | --- | --- |
| 1 | -1.137 | -1.440 | -0.491 | -0.823 | -1.095 | 0.486 | -0.077 | 0.546 | -1.137 | -1.440 |
| 2 | -0.101 | 1.393 | 1.377 | -0.260 | 1.746 | 1.058 | 0.861 | 0.917 | -0.101 | 1.393 |
| 3 | 0.753 | 1.184 | 0.597 | 1.821 | 0.912 | 1.180 | 0.183 | 1.116 | 0.753 | 1.184 |
| 4 | 0.803 | 1.710 | 1.165 | 1.208 | 1.513 | 0.408 | 1.131 | 0.464 | 0.803 | 1.710 |
| 5 | -0.044 | -0.985 | -0.500 | 0.220 | -0.528 | -2.330 | 0.399 | -2.121 | -0.044 | -0.985 |
| 6 | 1.269 | 0.836 | 1.256 | -0.026 | 0.998 | 1.269 | 2.006 | 1.350 | 1.269 | 0.836 |
| 7 | -1.330 | -1.523 | -0.550 | -0.435 | -0.244 | 0.256 | -0.725 | 0.056 | -1.330 | -1.523 |
| 8 | -0.851 | 0.891 | 1.102 | -0.434 | 0.495 | 0.972 | -0.649 | 0.439 | -0.851 | 0.891 |
| 9 | 1.012 | 0.409 | 1.770 | -0.791 | 0.622 | 0.478 | 0.486 | 1.289 | 1.012 | 0.409 |
| 10 | -1.496 | -0.493 | -1.160 | -1.826 | -0.566 | -1.563 | 0.357 | 0.812 | -1.496 | -0.493 |
| 11 | 0.642 | -1.203 | 0.445 | 0.221 | 0.165 | 0.840 | -0.312 | -0.125 | 0.642 | -1.203 |
| 12 | -0.210 | 0.474 | 0.759 | -0.263 | 1.338 | -0.224 | -0.480 | -1.042 | -0.210 | 0.474 |
| 13 | 1.596 | 0.245 | -1.256 | 0.481 | -1.902 | -0.106 | -0.568 | -1.523 | 1.596 | 0.245 |
| 14 | 0.866 | 0.893 | 0.506 | 0.983 | 1.092 | 0.239 | 1.175 | 0.039 | 0.866 | 0.893 |
| 15 | 0.414 | 0.557 | 1.348 | 0.165 | 0.465 | 0.482 | 0.906 | 0.077 | 0.414 | 0.557 |
| 16 | -0.087 | -0.355 | -0.123 | -0.858 | -0.706 | 0.659 | -0.456 | 0.422 | -0.087 | -0.355 |
| 17 | -2.118 | -0.825 | -1.740 | -0.734 | -2.438 | -0.103 | 1.181 | 0.953 | -2.118 | -0.825 |
| 18 | -1.568 | -1.037 | 0.463 | -0.451 | 1.060 | -1.370 | 0.716 | -1.913 | -1.568 | -1.037 |
| 19 | -0.308 | -0.223 | -0.181 | -0.054 | -0.790 | -0.488 | -1.037 | -0.563 | -0.308 | -0.223 |
| 20 | 1.208 | 1.232 | -0.192 | -0.575 | -0.512 | -0.175 | -0.995 | -0.346 | 1.208 | 1.232 |
| 21 | 0.482 | 0.766 | 0.160 | -1.202 | 0.170 | 0.950 | -1.933 | 0.948 | 0.482 | 0.766 |
| 22 | 0.843 | -1.118 | -1.514 | 1.582 | -0.049 | 1.106 | -1.509 | 0.774 | 0.843 | -1.118 |
| 23 | -1.400 | 0.798 | -1.135 | 0.622 | 0.623 | 1.218 | -0.835 | 1.172 | -1.400 | 0.798 |
| 24 | 0.630 | -1.411 | 0.624 | 0.619 | 0.041 | 0.910 | 1.280 | 1.408 | 0.630 | -1.411 |
| 25 | 0.275 | 1.403 | 1.285 | 1.936 | 0.686 | 1.086 | 0.050 | 0.753 | 0.275 | 1.403 |
| 26 | 1.103 | -0.727 | -0.192 | -0.862 | -0.198 | -0.536 | 1.284 | -0.536 | 1.103 | -0.727 |
| 27 | 0.215 | -0.404 | -0.886 | 1.031 | -0.642 | -0.284 | 0.569 | -0.255 | 0.215 | -0.404 |
| 28 | 0.407 | 1.448 | 0.755 | 0.621 | 0.430 | -0.326 | 0.574 | -0.882 | 0.407 | 1.448 |
| 29 | -1.333 | -0.241 | -0.937 | 0.800 | -0.688 | -0.793 | -1.022 | -0.873 | -1.333 | -0.241 |
| 30 | 0.803 | 0.514 | 0.484 | 0.515 | 0.796 | 0.828 | 1.639 | 0.605 | 0.803 | 0.514 |
| 31 | -1.201 | -1.388 | -0.610 | -0.067 | -0.926 | -1.117 | -1.139 | -0.736 | -1.201 | -1.388 |
| 32 | 2.120 | 2.254 | 1.469 | 0.903 | 1.480 | 1.042 | 1.379 | 1.143 | 2.120 | 2.254 |
| 33 | 0.033 | -0.246 | -0.126 | -0.095 | -0.154 | 0.372 | 0.120 | 0.338 | 0.033 | -0.246 |
| 34 | -0.155 | -0.688 | 0.468 | -1.579 | 0.775 | -1.774 | -0.727 | -0.882 | -0.155 | -0.688 |
| 35 | -0.055 | -0.347 | -0.677 | -2.053 | -0.993 | -0.066 | -0.088 | 0.242 | -0.055 | -0.347 |
| 36 | -0.303 | -0.584 | -0.151 | 1.296 | 0.717 | -0.735 | -0.195 | -0.990 | -0.303 | -0.584 |
| 37 | -0.388 | 0.046 | 0.183 | 0.419 | -0.121 | -1.633 | -0.553 | -1.669 | -0.388 | 0.046 |
| 38 | 0.623 | -0.097 | 0.180 | -0.244 | -0.118 | 0.828 | 0.402 | 1.170 | 0.623 | -0.097 |
