# Supplementary material for: The Accuracy of Survival Time Prediction for Patients with Glioma Is Improved by Measuring Mitotic Spindle Checkpoint Gene Expression
Source: PLoS One. 2011 Oct 12;6(10):e25631. doi: 10.1371/journal.pone.0025631 (PMC3192043; doi:10.1371/journal.pone.0025631)
Supplement: Information S4 — Screening all possible combinations of eight SAC genes to construct a linear model to predict grade. (DOC) [file pone.0025631.s004.doc]

**Supporting Information S4. Screening all possible combinations of eight SAC genes to construct a linear model to predict grade**

| score | genes |
| --- | --- |
| -7 | BUB1 + BUB1B + CDC20 |
| -7 | CDC20 + MAD2L1 |
| -6 | CDC20 + CENPE |
| -6 | BUB1B + CDC20 |
| -6 | BUB1B + CDC20 + MAD2L1 |
| -5 | BUB1B + CDC20 + MAD1L1 |
| -6 | BUB1B + BUB3 + CDC20 |
| -7 | BUB1 + CDC20 + CENPE |
| -7 | CDC20 + CENPE + MAD2L1 |
| -6 | BUB3 + CDC20 + CENPE |
| -6 | BUB1B + CDC20 + TTK |
| -7 | BUB1 + BUB1B + BUB3 + CDC20 |
| -7 | CDC20 + CENPE + TTK |
| -6 | BUB1B + BUB3 + CDC20 + MAD1L1 |
| -6 | BUB1B + BUB3 + CDC20 + MAD2L1 |
| -7 | BUB1 + BUB1B + CDC20 + MAD1L1 |
| -6 | BUB1B + CDC20 + CENPE + MAD1L1 |
| -4 | BUB1B + CDC20 + MAD1L1 + TTK |
| -7 | BUB1B + CDC20 + MAD1L1 + MAD2L1 |
| -6 | BUB1B + BUB3 + CDC20 + TTK |
| -7 | BUB1B + CDC20 + MAD2L1 + TTK |
| -7 | BUB1 + BUB1B + BUB3 + CDC20 + TTK |
| -6 | BUB1B + BUB3 + CDC20 + MAD1L1 + TTK |
| -7 | BUB1B + BUB3 + CDC20 + MAD1L1 + MAD2L1 |
| -6 | BUB1B + CDC20 + CENPE + MAD1L1 + MAD2L1 |
| -6 | BUB1B + CDC20 + CENPE + MAD1L1 + TTK |
| -7 | BUB1B + BUB3 + CDC20 + MAD2L1 + TTK |
| -7 | BUB1B + BUB3 + CDC20 + CENPE + MAD1L1 |
| -5 | BUB1 + BUB1B + CDC20 + MAD1L1 + TTK |
| -6 | BUB1 + BUB1B + BUB3 + CDC20 + MAD1L1 + TTK |
| -5 | BUB1B + CDC20 + MAD1L1 + MAD2L1 + TTK |
| -7 | BUB1B + BUB3 + CDC20 + MAD1L1 + MAD2L1 + TTK |
| -6 | BUB1 + BUB1B + CDC20 + MAD1L1 + MAD2L1 + TTK |
| -7 | BUB1 + BUB1B + CDC20 + CENPE + MAD1L1 + TTK |
| -7 | BUB1B + CDC20 + CENPE + MAD1L1 + MAD2L1 + TTK |

Score: Mistake number with model. n=38
