# Supplementary material for: The Accuracy of Survival Time Prediction for Patients with Glioma Is Improved by Measuring Mitotic Spindle Checkpoint Gene Expression
Source: PLoS One. 2011 Oct 12;6(10):e25631. doi: 10.1371/journal.pone.0025631 (PMC3192043; doi:10.1371/journal.pone.0025631)
Supplement: Information S5 — Leave-one-out cross-validation for prediction of WHO grade for 38 patients. (DOC) [file pone.0025631.s005.doc]

**Supporting Information S5. Leave-one-out cross-validation for prediction of WHO grade for 38 patients.**

| Intercept | BUB1B | CDC20 | MAD1L1 | TTK | Grade | Grade_predict | Difference |
| --- | --- | --- | --- | --- | --- | --- | --- |
| 0.61 | 0.13 | 0.29 | 0.06 | -0.03 | 2 | 2 | 0 |
| 0.61 | 0.13 | 0.29 | 0.06 | -0.03 | 3 | 3 | 0 |
| 0.61 | 0.13 | 0.29 | 0.06 | -0.03 | 3 | 3 | 0 |
| 0.62 | 0.13 | 0.28 | 0.06 | -0.03 | 2 | 2 | 0 |
| 0.60 | 0.13 | 0.33 | 0.06 | -0.05 | 1 | 1 | 0 |
| 0.62 | 0.13 | 0.29 | 0.06 | -0.03 | 3 | 3 | 0 |
| 0.61 | 0.13 | 0.29 | 0.06 | -0.03 | 2 | 2 | 0 |
| 0.61 | 0.13 | 0.28 | 0.06 | -0.02 | 3 | 3 | 0 |
| 0.61 | 0.12 | 0.29 | 0.05 | -0.03 | 3 | 3 | 0 |
| 0.62 | 0.19 | 0.22 | 0.06 | -0.03 | 1 | 2 | 1 |
| 0.62 | 0.12 | 0.30 | 0.06 | -0.03 | 2 | 2 | 0 |
| 0.61 | 0.15 | 0.27 | 0.05 | -0.02 | 2 | 2 | 0 |
| 0.62 | 0.09 | 0.32 | 0.04 | -0.03 | 1 | 1 | 0 |
| 0.61 | 0.13 | 0.29 | 0.06 | -0.03 | 2 | 2 | 0 |
| 0.61 | 0.13 | 0.29 | 0.06 | -0.03 | 2 | 2 | 0 |
| 0.62 | 0.12 | 0.30 | 0.06 | -0.03 | 2 | 2 | 0 |
| 0.61 | 0.11 | 0.30 | 0.08 | -0.03 | 2 | 2 | 0 |
| 0.61 | 0.13 | 0.29 | 0.06 | -0.03 | 1 | 1 | 0 |
| 0.62 | 0.12 | 0.29 | 0.05 | -0.03 | 1 | 1 | 0 |
| 0.61 | 0.13 | 0.28 | 0.06 | -0.02 | 2 | 2 | 0 |
| 0.61 | 0.13 | 0.28 | 0.06 | -0.03 | 3 | 3 | 0 |
| 0.61 | 0.12 | 0.29 | 0.06 | -0.04 | 3 | 3 | 0 |
| 0.61 | 0.13 | 0.29 | 0.06 | -0.03 | 3 | 3 | 0 |
| 0.62 | 0.15 | 0.28 | 0.05 | -0.02 | 2 | 3 | 1 |
| 0.61 | 0.12 | 0.29 | 0.06 | -0.04 | 3 | 3 | 0 |
| 0.62 | 0.12 | 0.29 | 0.06 | -0.04 | 1 | 1 | 0 |
| 0.60 | 0.12 | 0.30 | 0.07 | -0.05 | 2 | 1 | 1 |
| 0.62 | 0.11 | 0.29 | 0.06 | -0.03 | 1 | 1 | 0 |
| 0.62 | 0.13 | 0.28 | 0.05 | -0.02 | 1 | 1 | 0 |
| 0.61 | 0.13 | 0.29 | 0.06 | -0.03 | 3 | 3 | 0 |
| 0.62 | 0.13 | 0.28 | 0.06 | -0.03 | 1 | 1 | 0 |
| 0.61 | 0.13 | 0.29 | 0.06 | -0.03 | 3 | 3 | 0 |
| 0.60 | 0.13 | 0.28 | 0.06 | -0.03 | 3 | 2 | 1 |
| 0.61 | 0.13 | 0.29 | 0.06 | -0.03 | 1 | 1 | 0 |
| 0.61 | 0.13 | 0.28 | 0.06 | -0.02 | 2 | 2 | 0 |
| 0.62 | 0.13 | 0.28 | 0.06 | -0.02 | 1 | 1 | 0 |
| 0.61 | 0.13 | 0.30 | 0.06 | -0.03 | 1 | 1 | 0 |
| 0.61 | 0.12 | 0.29 | 0.06 | -0.03 | 3 | 3 | 0 |
