# Supplementary material for: The Accuracy of Survival Time Prediction for Patients with Glioma Is Improved by Measuring Mitotic Spindle Checkpoint Gene Expression
Source: PLoS One. 2011 Oct 12;6(10):e25631. doi: 10.1371/journal.pone.0025631 (PMC3192043; doi:10.1371/journal.pone.0025631)
Supplement: Information S6 — Correlation analysis between SAC genes and other factors in 38 glioma samples. (DOCX) [file pone.0025631.s006.docx]

**Supporting Information S6. Correlation analysis between SAC genes and other factors in 38 glioma samples**

|  |  |  |  |  |  |  |  |  |  |  |  |  |
| --- | --- | --- | --- | --- | --- | --- | --- | --- | --- | --- | --- | --- |
|  | BUB1 | CENPE | MAD2L1 | TTK | MAD1L1 | CDC20 | BUB3 | BUB1B | Survival | Ki_67_Perc | Mitotic_Score | WHO_Grade |
|  |  |  |  |  |  |  |  |  |  |  |  |  |
| BUB1 | NA | 0.48 | 0.45 | 0.31 | 0.33 | 0.39 | 0.29 | 0.18 | -0.13 | 0.22 | 0.18 | 0.32 |
| CENPE | 2.3E-03 | NA | 0.58 | 0.31 | 0.56 | 0.41 | 0.23 | 0.28 | -0.38 | 0.45 | 0.41 | 0.44 |
| MAD2L1 | 4.3E-03 | 1.6E-04 | NA | 0.14 | 0.78 | 0.33 | 0.41 | 0.22 | -0.26 | 0.32 | 0.36 | 0.36 |
| TTK | 5.7E-02 | 5.9E-02 | 3.9E-01 | NA | 0.32 | 0.29 | 0.12 | 0.01 | 0.06 | 0.25 | 0.15 | 0.18 |
| MAD1L1 | 4.4E-02 | 2.8E-04 | 9.3E-09 | 5.4E-02 | NA | 0.30 | 0.30 | 0.18 | -0.22 | 0.32 | 0.31 | 0.38 |
| CDC20 | 1.6E-02 | 9.9E-03 | 4.0E-02 | 7.8E-02 | 6.6E-02 | NA | 0.15 | 0.80 | -0.57 | 0.63 | 0.59 | 0.86 |
| BUB3 | 7.8E-02 | 1.7E-01 | 9.7E-03 | 4.6E-01 | 6.3E-02 | 3.8E-01 | NA | 0.26 | -0.26 | 0.33 | 0.41 | 0.14 |
| BUB1B | 2.7E-01 | 8.6E-02 | 2.0E-01 | 9.7E-01 | 2.7E-01 | 2.0E-09 | 1.2E-01 | NA | -0.81 | 0.78 | 0.72 | 0.80 |
| Survival | 4.2E-01 | 2.E-02 | 1.1E-01 | 7.2E-01 | 1.9E-01 | 2.1E-04 | 1.1E-01 | 5.1E-10 | NA | -0.73 | -0.72 | -0.63 |
| Ki-67 Li | 1.8E-01 | 4.3E-03 | 4.9E-02 | 1.3E-01 | 5.3E-02 | 2.0E-05 | 4.5E-02 | 6.8E-09 | 2.2E-07 | NA | 0.85 | 0.72 |
| MI | 2.7E-01 | 1.2E-02 | 2.6E-02 | 3.6E-01 | 6.2E-02 | 1.0E-04 | 1.1E-02 | 3.2E-07 | 2.7E-07 | 1.8E-11 | NA | 0.69 |
| WHO Grade | 5.3E-02 | 5.2E-03 | 2.7E-02 | 2.8E-01 | 1.8E-02 | 4.0E-12 | 4.0E-01 | 1.7E-09 | 2.3E-05 | 2.7E-07 | 1.7E-06 | NA |

Pearson’s correlation coefficients are above the diagonal and *p* values are below. Coefficients better than 0.67 and p <0.0001 are indicated in red.
