# Supplementary material for: The Accuracy of Survival Time Prediction for Patients with Glioma Is Improved by Measuring Mitotic Spindle Checkpoint Gene Expression
Source: PLoS One. 2011 Oct 12;6(10):e25631. doi: 10.1371/journal.pone.0025631 (PMC3192043; doi:10.1371/journal.pone.0025631)
Supplement: Information S7 — Screening all possible combinations of eight SAC genes to construct a linear model to predict survival time. (DOC) [file pone.0025631.s007.doc]

**Supporting Information S7. Screening all possible combinations of eight SAC genes to construct a linear model to predict survival time**

| score | genes |
| --- | --- |
| -49.4 | BUB1 + BUB1B |
| -46.4 | BUB1 + BUB1B + CENPE |
| -45.9 | BUB1 + BUB1B + MAD2L1 |
| -49.6 | BUB1B + TTK |
| -49.0 | BUB1 + BUB1B + TTK |
| -48.0 | BUB1 + BUB1B + BUB3 + CENPE |
| -47.7 | BUB1 + BUB1B + CDC20 + CENPE |
| -46.7 | BUB1 + BUB1B + CENPE + MAD2L1 |
| -46.8 | BUB1 + BUB1B + CDC20 + MAD2L1 |
| -48.7 | BUB1 + BUB1B + BUB3 + MAD2L1 |
| -47.4 | BUB1B + CENPE + TTK |
| -48.9 | BUB1 + BUB1B + MAD1L1 + TTK |
| -49.5 | BUB1 + BUB1B + MAD1L1 + MAD2L1 |
| -44.2 | BUB1 + BUB1B + CENPE + TTK |
| -45.2 | BUB1 + BUB1B + MAD2L1 + TTK |
| -49.2 | BUB1 + BUB1B + BUB3 + CENPE + MAD2L1 |
| -47.8 | BUB1 + BUB1B + BUB3 + MAD2L1 + TTK |
| -45.8 | BUB1 + BUB1B + BUB3 + CENPE + TTK |
| -49.9 | BUB1B + CENPE + MAD2L1 + TTK |
| -47.8 | BUB1 + BUB1B + CDC20 + CENPE + TTK |
| -47.2 | BUB1 + BUB1B + CENPE + MAD1L1 + TTK |
| -45.0 | BUB1 + BUB1B + CENPE + MAD2L1 + TTK |
| -48.2 | BUB1 + BUB1B + CDC20 + MAD2L1 + TTK |
| -46.9 | BUB1 + BUB1B + CDC20 + CENPE + MAD2L1 |
| -48.9 | BUB1 + BUB1B + BUB3 + CENPE + MAD1L1 + TTK |
| -47.7 | BUB1 + BUB1B + CDC20 + CENPE + MAD2L1 + TTK |
| -47.7 | BUB1 + BUB1B + BUB3 + CENPE + MAD2L1 + TTK |
| -49.9 | BUB1 + BUB1B + BUB3 + CDC20 + CENPE + MAD2L1 |
| -48.3 | BUB1 + BUB1B + MAD1L1 + MAD2L1 + TTK |
| -48.7 | BUB1 + BUB1B + CENPE + MAD1L1 + MAD2L1 + TTK |

n=38
