# Supplementary material for: The Accuracy of Survival Time Prediction for Patients with Glioma Is Improved by Measuring Mitotic Spindle Checkpoint Gene Expression
Source: PLoS One. 2011 Oct 12;6(10):e25631. doi: 10.1371/journal.pone.0025631 (PMC3192043; doi:10.1371/journal.pone.0025631)
Supplement: Information S8 — Leave-one-out cross-validation of prediction of survival time for 34 deceased patients using the four-gene model. (DOC) [file pone.0025631.s008.doc]

**Supporting Information S8. Leave-one-out cross-validation of prediction of survival time for 34 deceased patients using the four-gene model**

| (Intercept) | BUB1 | BUB1B | CENPE | MAD2L1 | Survival_predict | Survival | Difference | Grade |
| --- | --- | --- | --- | --- | --- | --- | --- | --- |
| 20.71 | 5.40 | -15.70 | -3.45 | -5.38 | 20.71 | 8.2 | 4.338993 | III |
| 20.54 | 5.82 | -15.97 | -3.38 | -5.73 | 20.54 | 3.7 | 2.246243 | IV |
| 20.68 | 5.71 | -16.07 | -3.16 | -5.64 | 20.68 | 6.5 | 2.760629 | IV |
| 20.66 | 5.53 | -14.51 | -3.04 | -5.80 | 20.66 | 10.6 | 7.36047 | III |
| 20.70 | 5.67 | -16.35 | -3.26 | -5.60 | 20.70 | 45.1 | 2.047594 | II |
| 20.61 | 5.66 | -15.69 | -3.30 | -5.52 | 20.61 | 2.6 | 1.582849 | IV |
| 20.41 | 6.35 | -16.37 | -3.29 | -6.36 | 20.41 | 10.1 | 7.395605 | IV |
| 20.55 | 5.63 | -15.88 | -3.23 | -5.77 | 20.55 | 3.1 | 1.669827 | IV |
| 20.64 | 5.57 | -15.97 | -3.25 | -5.66 | 20.64 | 7.2 | 1.706508 | II |
| 20.17 | 4.87 | -13.37 | -2.73 | -5.40 | 20.17 | 40.3 | 6.460232 | III |
| 20.83 | 5.28 | -15.43 | -3.18 | -4.77 | 20.83 | 15.9 | 8.358211 | III |
| 20.83 | 6.11 | -14.95 | -3.18 | -6.10 | 20.83 | 15.9 | 10.66741 | III |
| 20.52 | 5.77 | -15.94 | -3.14 | -5.75 | 20.52 | 21.1 | 2.832118 | III |
| 20.46 | 5.70 | -16.05 | -3.23 | -5.84 | 20.46 | 13.8 | 5.078654 | III |
| 20.62 | 5.35 | -15.47 | -3.24 | -5.69 | 20.62 | 6.2 | 2.147774 | III |
| 20.27 | 5.77 | -16.99 | -3.15 | -5.41 | 20.27 | 40.2 | 12.17203 | II |
| 20.56 | 5.64 | -15.62 | -3.31 | -5.56 | 20.56 | 22.7 | 0.467951 | III |
| 20.56 | 5.64 | -15.69 | -3.30 | -5.57 | 20.56 | 10.9 | 0.584089 | IV |
| 20.38 | 5.00 | -15.43 | -3.10 | -5.16 | 20.38 | 20.3 | 6.221816 | IV |
| 20.40 | 5.46 | -15.55 | -3.57 | -5.27 | 20.40 | 8.6 | 4.631349 | IV |
| 20.61 | 5.35 | -13.01 | -4.36 | -5.24 | 20.61 | 5.5 | 9.519593 | III |
| 20.49 | 5.69 | -14.86 | -3.34 | -5.60 | 20.49 | 11.9 | 1.075694 | IV |
| 20.55 | 5.63 | -15.69 | -3.27 | -5.60 | 20.55 | 27.2 | 0.835545 | II |
| 20.57 | 5.65 | -15.62 | -3.27 | -5.59 | 20.57 | 21.8 | 0.02814 | III |
| 20.53 | 5.71 | -15.95 | -3.45 | -5.53 | 20.53 | 30.5 | 2.382398 | II |
| 20.85 | 5.66 | -15.27 | -3.26 | -5.97 | 20.85 | 27.8 | 7.723123 | II |
| 20.41 | 5.90 | -18.57 | -3.00 | -4.62 | 20.41 | 33.6 | 16.06612 | IV |
| 20.61 | 5.57 | -15.33 | -3.33 | -5.55 | 20.61 | 27.5 | 1.529186 | II |
| 20.61 | 5.77 | -15.71 | -3.23 | -5.61 | 20.61 | 8.9 | 1.309018 | IV |
| 20.61 | 5.70 | -15.56 | -3.28 | -5.59 | 20.61 | 17.9 | 1.079141 | IV |
| 20.43 | 5.57 | -14.99 | -3.20 | -5.81 | 20.43 | 29.6 | 2.478969 | II |
| 20.62 | 5.67 | -15.10 | -3.23 | -5.77 | 20.62 | 18.7 | 3.063043 | III |
| 20.57 | 5.65 | -15.62 | -3.27 | -5.59 | 20.57 | 40.5 | 0.011282 | II |
| 20.70 | 5.97 | -15.23 | -3.49 | -5.69 | 20.70 | 8.5 | 5.246843 | IV |
