# Supplementary material for: The Accuracy of Survival Time Prediction for Patients with Glioma Is Improved by Measuring Mitotic Spindle Checkpoint Gene Expression
Source: PLoS One. 2011 Oct 12;6(10):e25631. doi: 10.1371/journal.pone.0025631 (PMC3192043; doi:10.1371/journal.pone.0025631)
Supplement: Information S9 — Leave-one-out cross-validation of prediction of survival time for 34 deceased patients using the one gene BUB1B model. (DOC) [file pone.0025631.s009.doc]

**Supporting Information S9. Leave-one-out cross-validation of prediction of survival time for 34 deceased patients using the one gene BUB1B model.**

| (Intercept) | BUB1B | Survival  prediction | Survival | Difference | Grade |
| --- | --- | --- | --- | --- | --- |
| 20.30 | -11.18 | 14.84 | 8.2 | 6.64 | III |
| 20.30 | -11.07 | 10.69 | 3.7 | 6.99 | IV |
| 20.16 | -11.20 | 8.17 | 6.5 | 1.67 | IV |
| 20.26 | -11.21 | 15.72 | 10.6 | 5.12 | III |
| 20.13 | -11.28 | 45.35 | 45.1 | 0.25 | II |
| 20.19 | -11.13 | 5.62 | 2.6 | 3.02 | IV |
| 20.29 | -11.21 | 16.04 | 10.1 | 5.94 | IV |
| 20.19 | -11.13 | 6.32 | 3.1 | 3.22 | IV |
| 20.24 | -11.16 | 11.76 | 7.2 | 4.56 | II |
| 19.53 | -11.01 | 21.70 | 40.3 | 18.60 | III |
| 20.82 | -12.18 | 34.63 | 15.9 | 18.73 | III |
| 20.26 | -11.29 | 20.60 | 15.9 | 4.70 | III |
| 20.09 | -11.25 | 19.98 | 21.1 | 1.12 | III |
| 20.19 | -11.24 | 16.12 | 13.8 | 2.32 | III |
| 20.22 | -11.15 | 10.15 | 6.2 | 3.95 | III |
| 19.66 | -10.85 | 26.64 | 40.2 | 13.56 | II |
| 20.20 | -11.31 | 24.97 | 22.7 | 2.27 | III |
| 20.10 | -11.29 | 9.96 | 10.9 | 0.95 | IV |
| 19.90 | -11.45 | 11.64 | 20.3 | 8.66 | IV |
| 20.09 | -11.31 | 7.34 | 8.6 | 1.26 | IV |
| 20.10 | -11.30 | 4.64 | 5.5 | 0.86 | III |
| 20.13 | -11.25 | 12.26 | 11.9 | 0.36 | IV |
| 20.12 | -11.26 | 27.06 | 27.2 | 0.14 | II |
| 20.19 | -11.30 | 23.91 | 21.8 | 2.11 | III |
| 20.14 | -11.29 | 31.08 | 30.5 | 0.58 | II |
| 20.24 | -11.41 | 31.20 | 27.8 | 3.40 | II |
| 19.57 | -11.56 | 13.22 | 33.6 | 20.38 | IV |
| 20.19 | -11.34 | 29.50 | 27.5 | 2.00 | II |
| 20.09 | -11.30 | 7.68 | 8.9 | 1.22 | IV |
| 20.09 | -11.27 | 16.99 | 17.9 | 0.91 | IV |
| 20.18 | -11.33 | 31.17 | 29.6 | 1.57 | II |
| 20.10 | -11.26 | 18.10 | 18.7 | 0.60 | III |
| 19.79 | -10.84 | 31.49 | 40.5 | 9.01 | II |
| 20.09 | -11.30 | 7.38 | 8.5 | 1.12 | IV |
