# Supplementary material for: The Accuracy of Survival Time Prediction for Patients with Glioma Is Improved by Measuring Mitotic Spindle Checkpoint Gene Expression
Source: PLoS One. 2011 Oct 12;6(10):e25631. doi: 10.1371/journal.pone.0025631 (PMC3192043; doi:10.1371/journal.pone.0025631)
Supplement: Information S10 — Examples of immunohistochemical staining. (DOC) [file pone.0025631.s010.doc]

**Supporting Information S10.** **Examples of immunohistochemical staining**


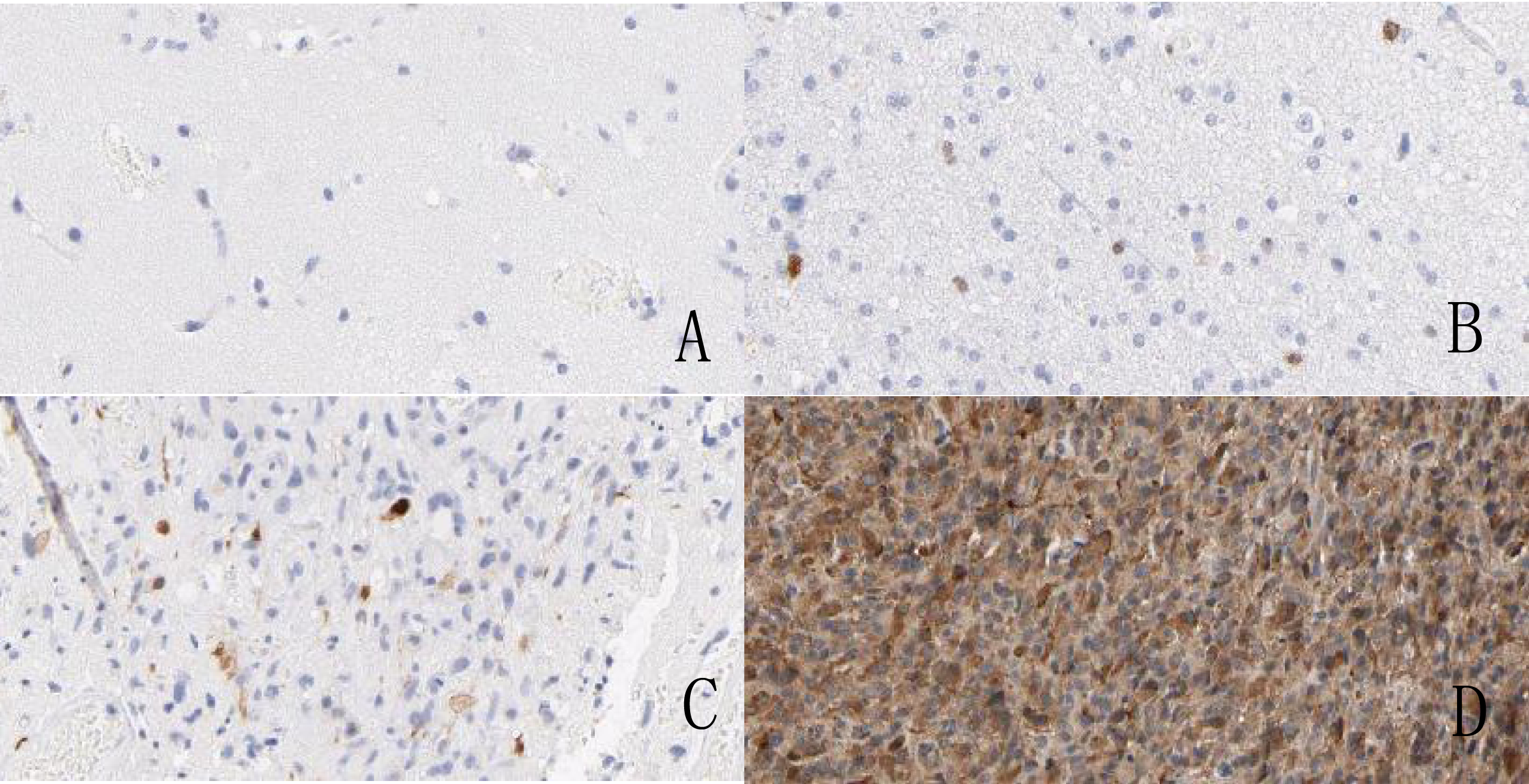


IHC of gliomas were scored as follows: A: Negative, B: Weak, C: Moderate, D: Strong (taken from <http://www.proteinatlas.org/>). Panels A-C: CDC20, D: BUB1B.
